# The diagnostic role of resting myocardial blood flow in STEMI patients after revascularization

**DOI:** 10.3389/fcvm.2024.1364772

**Published:** 2024-03-20

**Authors:** Ming Yan, Hua Shang, Xiaorui Guo, Luping Hao, Shuang Hou, Hongming Zheng

**Affiliations:** ^1^ Department of Nuclear Medicine, The Second Hospital of Hebei Medical University, Shijiazhuang, China; ^2^ Department of Electronic Science and Technology, School of Electronic and Information Engineering, Beijing Jiaotong University, Beijing, China

**Keywords:** myocardial perfusion imaging (MPI), myocardial blood flow (MBF), STEMI (myocardial infarction), post-reperfusion therapy, CZT-SPECT

## Abstract

**Background:**

The value of semiquantitative resting myocardial perfusion imaging (MPI) in coronary artery disease (CAD) is limited. At present, quantitative MPI can be performed by a new cadmium zinc tellurium single-photon emission computed tomography (CZT-SPECT) scan. The quantitative index of resting myocardial blood flow (MBF) has received little attention, and its manifestations and clinical value in the presence of unstable coronary blood flow have not been clarified.

**Purpose:**

In patients with ST-segment elevation myocardial infarction (STEMI), whether resting MBF can provide additional value of blood flow than semi-quantitative resting MPI is not sure. We also explored the influencing factors of resting MBF.

**Methods:**

This was a retrospective clinical study. We included 75 patients with STEMI in the subacute phase who underwent resting MPI and dynamic scans after reperfusion therapy. General patient information, STEMI-related data, MPI, gated MPI (G-MPI), and resting MBF data were collected and recorded. According to the clinically provided culprit vessels, the resting MBF was divided into ischemic MBF and non-ischemic MBF. The paired Wilcoxon signed-rank test was used for resting MBF. The receiver operating characteristic (ROC) curves were used to determine the optimal threshold for ischemia, and multiple linear regression analysis was used to analyze the influencing factors of resting MBF.

**Results:**

There was a statistically significant difference between the ischemic MBF and non-ischemic MBF [0.59 (0.47–0.72) vs. 0.76 (0.64–0.93), *p* < 0.0001]. The ROC curve analysis revealed that resting MBF could identify ischemia to a certain extent, with a cutoff value of 0.5975, area under the curve (AUC) = 0.666, sensitivity = 55.8%, and specificity = 68.7%. Male sex and summed rest score (SRS) were influencing factors for resting MBF.

**Conclusion:**

To a certain extent, resting MBF can suggest residual ischemia after reperfusion therapy in patients with STEMI. There was a negative correlation between male sex, SRS, and ischemic MBF. A lower resting MBF may be associated with more severe myocardial ischemia.

## Introduction

ST-segment elevation myocardial infarction (STEMI) is an acute severe aspect of coronary artery disease (CAD), commonly occurring as an acute myocardial infarction (AMI) (1, 2). Notably, myocardial perfusion imaging (MPI) can assess myocardial ischemia due to myocardial infarction. With the development of imaging technology and the clinical application of cyclotrons, MPI has entered the era of absolute quantification (3, 4). Moreover, recent studies have supported the consistency of the quantitative parameters between cadmium zinc tellurium single-photon emission computed tomography (CZT-SPECT) and positron emission tomography (PET) (4, 5). Quantitative indicators of myocardial blood flow (MBF) and myocardial flow reserve (MFR) reflect whole coronary perfusion and provide additional value for patients with CAD, especially for those with balanced ischemia and ischemia and no obstractive coronary artery disease (INOCA) (6, 7).

In patients with STEMI, timely reperfusion therapy is beneficial for salvaging ischemic myocardium (8). Consequently, most patients cannot complete MPI during the acute stage. Semiquantitative MPI, as a non-invasive, true response to myocardial perfusion, has been widely applied to subacute and long-term AMI and has been confirmed to have prognostic value (9–12). Moreover, some studies have utilized MBF and MFR to perform a hemodynamic analysis and prognostic prediction for patients after treatment for AMI (13, 14).

Our study investigated using safer and simpler quantitative resting MPI to evaluate patients with early STEMI. We attempted to analyze whether resting MBF made sense in early STEMI and focused on whether there was a difference between ischemia-related MBF and non-ischemia-related MBF. Furthermore, we explored the optimal threshold for ischemia and analyzed the connection between clinical characteristics and resting MBF.

## Materials and methods

### Study design and patients

This was a single-center, retrospective study with a small sample size. We collected data from the Second Hospital of Hebei Medical University between May 2021 and April 2023.

The inclusion criteria for patients were as follows: (1) had primary STEMI, which was diagnosed by experienced cardiologists according to guidelines (1); and (2) underwent reperfusion treatment, including pharmacological thrombolysis therapy and percutaneous coronary intervention (PCI) on infarction-related vessels. Cardiologists determined culprit vessels through patients' symptoms, electrocardiogram, and coronary angiography; (3) all participants completed quantitative resting MPI within 14 days after reperfusion treatment. The exclusion criteria were as follows: (1) patients with reinfarction; (2) patients who did not receive reperfusion treatment in the acute stage; (3) patients whose clinical information was missing, such as resting heart rate and blood pressure; (4) patients did not undergo a resting dynamic scan; and (5) patients whose imaging did not meet the clinical diagnosis.

**Figure 1:**
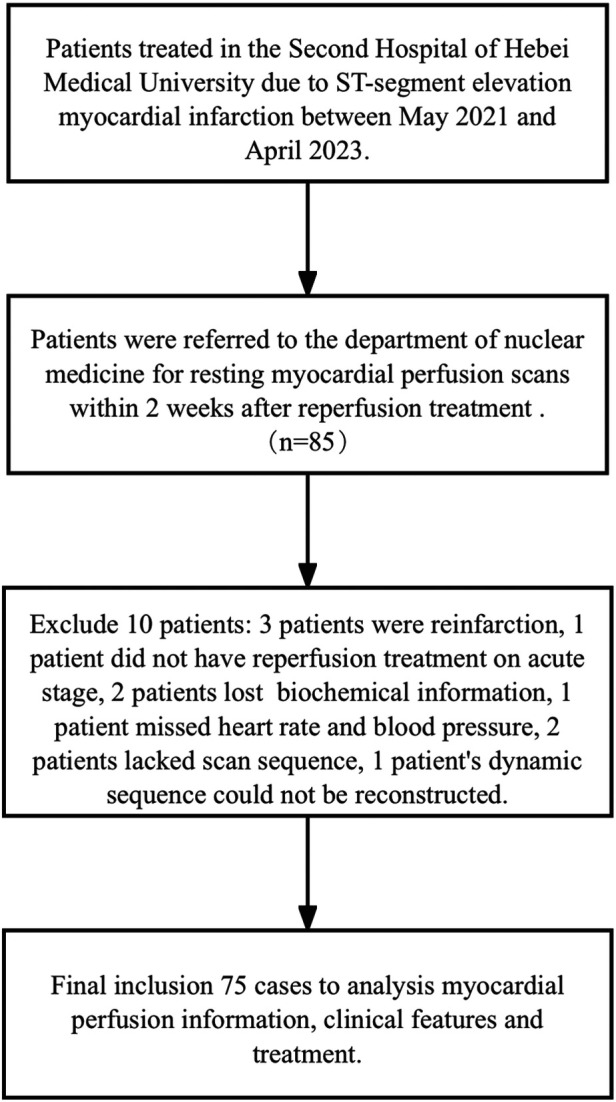
Flow chart of study inclusion and exclusion.

All patients undergoing MPI provided informed consent. Ultimately, 75 patients were included and 10 were excluded (Figure 1). Among those excluded, three patients had reinfarction, one did not receive reperfusion treatment in the acute stage, two lost biochemical information, one was missing heart rate and blood pressure, two lacked a scan sequence, and one dynamic sequence could not be reconstructed.

### CZT-SPECT acquisition

**Figure 2:**
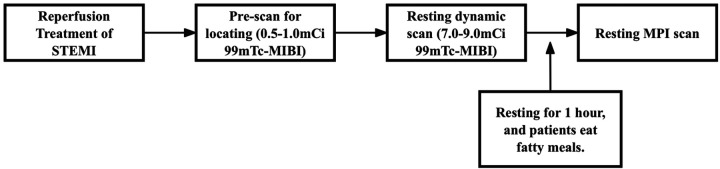
Resting MPI and resting MBF process in the subacute phase of STEMI.

Resting dynamic and perfusion scanning was performed on D-SPECT (Spectrum Dynamics, Caesarea, Israel). D-SPECT is a commercially available dedicated cardiac CZT-SPECT system with nine cadmium zinc telluride detection columns. The acquisition procedures and reconstruction parameters were the same as in previous studies (15). The specific imaging process is shown in Figure 2.

Patients were fasted before the examination and a fatty meal was prepared. Technicians conducted quality checks on imaging devices before imaging, while nurses established intravenous access for patients. Patients were made aware of the need to lie on the examination bed, and nuclear medicine physicians measured their blood pressure and heart rate 2–3 times while the patients were calm.

A prescan injection of 0.5–1.0 mCi ^99m^Tc-sestamibi (^99m^Tc-MIBI) was administered for cardiac localization, followed by a 7.0–9.0 mCi 2 mL bolus injected at 1 mL/s using a high-pressure syringe pump (ACIST Medical Systems). A 6-min CZT SPECT dynamic imaging acquisition was started at the same time as the bolus injection. Patients then rested for an interval of 1 h, consuming a fatty meal to promote hepatobiliary clearance and reduce infracardiac activity. After 1 h, the tracer was stably distributed on the myocardium, and the resting MPI was performed in both the supine and upright positions.

### Image reconstruction and image postprocessing

Both resting dynamic and perfusion sequences were checked for quality and clinical diagnostic requirements before they were reconstructed. Qualified scan sequences were reconstructed, and MBF was measured using QGS-QPS postprocessing commercial software (Cedars-Sinai Medical Center, Los Angeles, CA, USA). Before obtaining SPECT parameters, the endocardial and epicardial contouring and number of boluses were assessed. Dynamic images were corrected by motion correction (MC) and rate pressure product (RPP) correction to obtain the resting MBF. If the product of the resting heart rate and resting systolic blood pressure was greater than 10,000, the resting MBF equaled the product of heart rate and blood pressure divided by 10,000. Quantitative blood flow values were also calculated using the Renkin-Crone formula (16). Postprocessing software was also used to analyze the perfusion images to obtain semiquantitative perfusion parameters and G-MPI results.

The left ventricular bullseye was divided into 17 segments according to the American Heart Association (AHA) (17), each segment with an independent resting MBF. The resting MBF is divided into ischemic MBF and non-ischemic MBF. The ischemic area is dominated by the culprit vessels, and the non-ischemic area is governed by the non-culprit vessels. The segments dominated by the three branches of the coronary artery are prescribed by the AHA. The ischemic MBF is equal to the average resting MBF of each segment of the culprit vascular domination, and the non-ischemic MBF is equal to average resting MBF of each segment of the non-culprit vascular domination. All resting MBFs were measured independently by two physicians, each with at least 10 years of experience in nuclear medicine.

### Clinical feature data collection

All patients with STEMI were treated with reperfusion therapy and in-hospital medication according to guidelines (1, 18). All general and clinical information came from the hospital's medical records system and standard questionnaires from the Department of Nuclear Medicine. This information included sex, age, risk factors, onset and treatment of STEMI, biochemical indicators during hospitalization, electrocardiogram, CAG, PCI surgical records, and discharge diagnosis. Due to the precision of the cTnI detection method, the peak cTnI was classified into five grades: 0.04–25.00 = grade 1; 25.00–50.00 = grade 2; 50.00–75.00 = grade 3; 75.00–100.00 = grade 4; and >100.00 = grade 5.

### Statistical analyses

Continuous variables were shown as mean ±** **SD or median (interquartile range). Categorical variables were expressed as percentages. We also used the intraclass correlation coefficient (ICC) to test the consistency of the resting MBF. The resting MBF was the average of the two. The Wilcoxon signed-rank test of two paired samples was used for ischemic MBF and non-ischemic MBF. The resting MBF was analyzed using the receiver operating characteristic (ROC) curve, and the Youden index was calculated to obtain the cutoff value. Multiple linear regression was performed to examine the influence of ischemic, non-ischemic MBF. SPSS (version 26.0) and Prism (version 10.0) software were used. A *p*-value <0.05 was considered to indicate statistical significance or relevance.

## Results

In total, 75 patients were finally included, and the mean time from revascularization to CZT-SPECT was 7.1 days. The patients' sex, age, body mass index, and risk factors were collected (Table 1). The information about STEMI is summarized in Table 2, including electrocardiogram, CAG, thrombolysis in myocardial infarction (TIMI), flow, clinical features, treatment history, Killip grading, and biochemical indices. The summed rest score (SRS), total perfusion defect (TPD), end-diastolic volume (EDV), end-systolic volume (ESV), and left ventricular ejection fraction (LVEF) data are shown in Table 3.

**Table 1:** General information.

Variable	All subjects
Number of subjects	75
Sex (male)	67 (89.33%)
Age (year)	56.27 ± 1.48
Body mass index (BMI)	26.74 ± 0.43
Risk of CAD	
Hypertension	44 (58.67%)
Hyperlipidemia	46 (61.33%)
Diabetes	13 (17.33%)
Cerebrovascular diseases	8 (10.67%)
History of smoking	40 (53.33%)
Family history	10 (13.33%)

**Table 2:** Information about STEMI.

ST-segment elevation ECG leads	
V1-V6	34 (45.33%)
II, III, avF	41 (54.67%)
V7-V9, V3R-V5R	17 (22.67%)
I, avL	8 (10.67%)
Infarction-related wall of the chamber	
Anterior	30 (40.00%)
Septal	3 (4.00%)
Lateral	2 (2.67%)
Inferior	41 (54.67%)
Right ventricle	13 (17.33%)
Infarcted vessels	
Left main (LM)	0 (0%)
Left artery descending (LAD)	33 (44.00%)
Left circumflex (LCX)	3 (4.00%)
Right coronary artery (RCA)	39 (52.00%)
With multivessel coronary lesions	
2-vessels	20 (26.67%)
3-vessels	38 (50.67%)
Pre-infarction symptoms	
None	34 (45.33%)
Unprovoked chest tightness or chest pain	33 (44.00%)
Chest tightness or chest pain after exertion	8 (10.67%)
Killip grade	
I	60 (80.00%)
II	13 (17.33%)
III	0 (0%)
IV	2 (2.67%)
Diagnosis and treatment process	
Drug therapy (antiplatelet and anticoagulant agents)	69 (92.00%)
Time to drug therapy (h)	2.50 [1.00–4.50]
Thrombolytic therapy	46 (61.33%)
Time from onset to thrombolysis (h)	2.125 [1.50–3.875]
Percutaneous coronary intervention and/or Balloon	63 (84.00%)
Time to cath lab (h)	11.50 [7.09–18.00]
Slow flow during revascularization	8 (10.67%)
Post-revascularization TIMI	
2 grade 3 grade	2 (2.67%) 73 (97.33%)
Biochemical indicators	
Peak pro-BNP (pg/mL)	512.00 [129.00–1,249.00]
The grade of Peak cTnI (ng/mL)	
0.04–25.00	8 (10.67%)
25.00–50.00	2 (2.67%)
50.00–75.00	8 (10.67%)
75.00–100.00	8 (10.67%)
>100.00	49 (65.33%)
Time to SPECT (day)	7.00 [6.00–8.00]

**Table 3:** MPI and G-MPI results.

MPI results	
SRS	11.41 ± 1.16
TPD (%)	16.48 ± 1.72
G-MPI results	
EDV (mL)	112.61 ± 4.39
ESV (mL)	54.64 ± 4.00
EF (%)	54.92 ± 1.70

**Figure 3:**
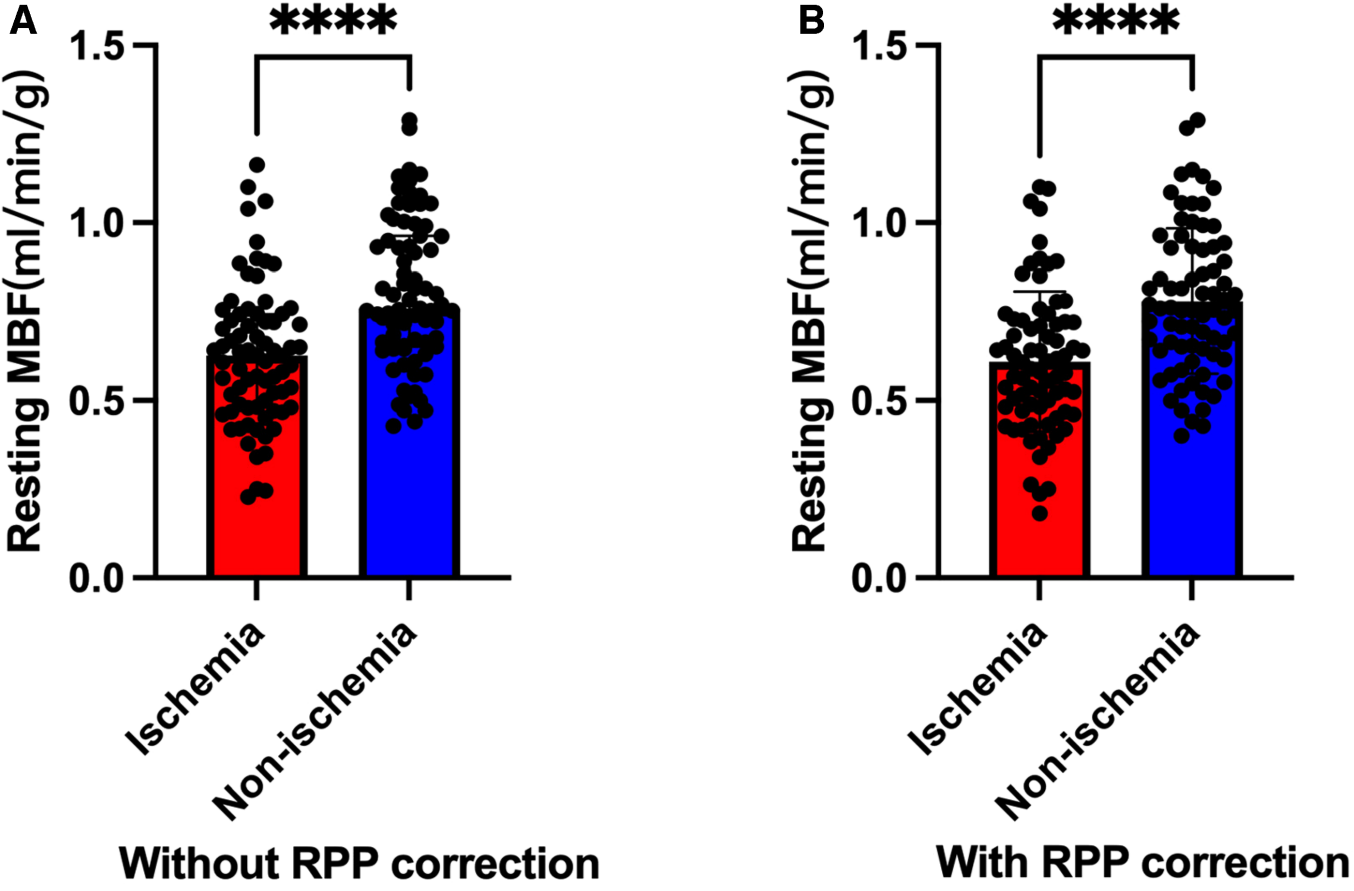
Ischemic MBF vs. non-ischemic MBF (****: *P* < 0.0001). (**A**) Without RPP correction. (**B**) With RPP correction.

**Figure 4:**
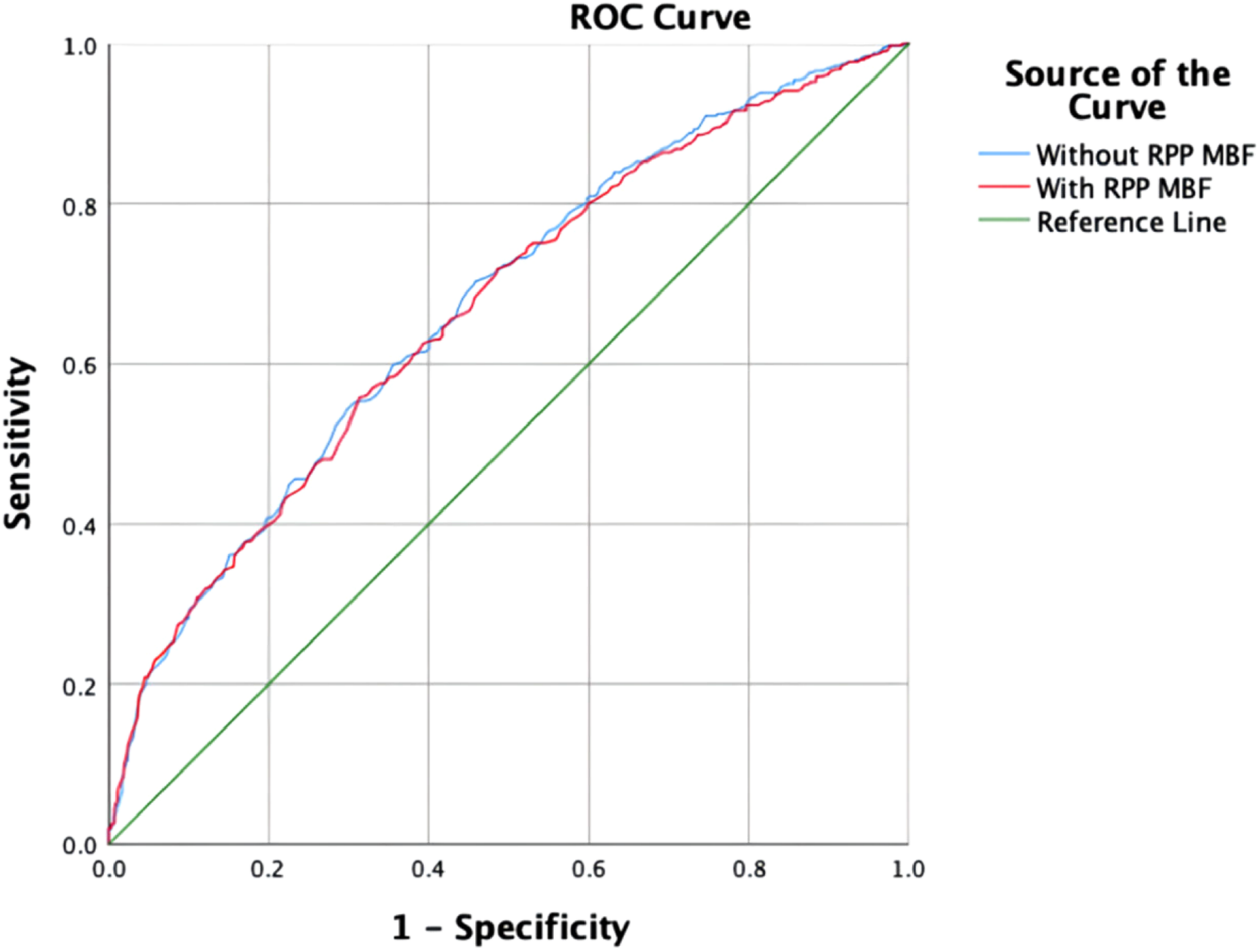
ROC curve of the resting MBF.

The blinded resting MBF of the ICC was good (ICC = 0.795). As shown in Figure 3 and Table 4, the resting MBF differed significantly (*p* < 0.0001) in ischemia- and non–ischemia-related areas. The ischemic MBF was lower than the non-ischemic MBF: the resting MBF with RPP was 0.59 (0.47–0.72) vs. 0.76 (0.64–0.93), and the resting MBF without RPP was 0.63 (0.48–0.74) vs. 0.76 (0.65–0.97). The ROC curve analysis suggested (Figure 4, Table 5) that the AUC was 0.666 for RPP (sensitivity = 55.8%, specificity = 68.7%) and the cutoff value was 0.5975; the AUC was 0.671 without RPP (sensitivity = 55.1%, specificity = 69.4%) and the cut-off value was 0.6175.

**Table 4:** Resting MBF in the subacute phase of STEMI.

Resting MBF (mL/min/g)	Ischemic MBF	Non-ischemic MBF	*P*-value
Resting MBF with RPP	0.59 [0.47–0.72]	0.76 [0.64–0.93]	<0.0001
Resting MBF without RPP	0.63 [0.48–0.74]	0.76 [0.65–0.97]	<0.0001

**Table 5:** ROC analysis of the resting MBF.

	Cutoff value	AUC value	Sensitivity	Specificity
Resting MBF with RPP	0.5975	0.666	0.558	0.687
Resting MBF without RPP	0.6175	0.671	0.551	0.694

On multiple linear regression (Table 6), the ischemic MBF was inversely correlated with male sex and SRS (with RPP: *β* = −0.336, *p* = 0.009; *β* = −1.240, *p* = 0.019, respectively; without RPP: *β* = −0.312, *p* = 0.017; *β* = −1.148, *p* = 0.034, respectively). The non-ischemic MBF was not associated with any factor.

**Table 6:** Multivariate analysis of the correlation between patients’ characteristics and resting MBF.

Variable	With RPP correction				Without RPP correction			
	Ischemic MBF		Non-ischemic MBF		Ischemic MBF		Non-ischemic MBF	
	Coefficient	*P*-value	Coefficient	*P*-value	Coefficient	*P*-value	Coefficient	*P*-value
Sex (male)	−0.336	0.009	−0.158	0.253	−0.312	0.017	−0.134	0.342
Age	0.128	0.392	0.239	0.153	0.120	0.439	0.196	0.250
Smoking	0.140	0.293	0.026	0.860	0.153	0.265	0.031	0.838
Diabetes	−0.180	0.115	0.012	0.924	−0.151	0.197	−0.031	0.811
Hypertension	−0.086	0.482	0.140	0.304	−0.068	0.592	0.191	0.172
Hyperlipidemia	0.204	0.100	0.189	0.168	0.217	0.090	0.190	0.174
Cerebrovascular diseases	−0.068	0.592	−0.195	0.168	−0.032	0.805	−0.154	0.283
Family history	0.000	0.998	0.010	0.938	−0.028	0.808	−0.030	0.811
Time from symptom of STEMI to reperfusion	−0.026	0.818	−0.058	0.647	−0.020	0.861	−0.056	0.664
Slow flow during revascularization	0.068	0.674	0.183	0.257	0.051	0.749	0.153	0.340
Postrevascularization TIMI flow	−0.087	0.591	0.021	0.895	−0.138	0.390	−0.049	0.761
Killip grade	−0.011	0.919	−0.047	0.701	0.003	0.979	−0.008	0.946
Peak BNP	0.018	0.895	−0.070	0.645	−0.032	0.821	−0.148	0.339
Peak cTnI	0.029	0.859	0.159	0.382	−0.002	0.990	0.114	0.539
SRS	−1.240	0.019	0.100	0.862	−1.148	0.034	0.467	0.424
TPD	0.544	0.258	−0.395	0.459	0.424	0.391	−0.709	0.195
EF	0.015	0.971	0.113	0.805	−0.105	0.804	−0.056	0.062
EDV	0.458	0.316	0.649	0.203	0.439	0.351	0.659	0.252
ESV	0.000	1.000	−0.497	0.504	−0.039	0.954	−0.80	0.445

## Discussion

Time is important for saving ischemic myocardium, and most patients with STEMI cannot complete MPI in the acute phase and stress test shortly after their surgery. Due to the self-regulation of the coronary arteries, the resting MBF has a large range of changes in homeostasis and its application value is limited (19). Thus, our study explored the value of resting MBF in patients with STEMI.

### Ischemia- and non–ischemia-related resting MBF

In our study, there was a difference between ischemic MBF and non-ischemic MBF after reperfusion treatment for patients with STEMI. We found that ischemic MBF was lower than non-ischemic MBF. In addition, the resting MBF in ischemic areas after reperfusion therapy was still low. Thus, we thought that STEMI reperfusion therapy solves the problem of epicardial blood vessel patency. However, why is the blood flow low in the area dominated by culprit vessels after treatment? This may be due to the recovery of coronary blood flow after treatment for STEMI, which requires a certain amount of time, as well as the impairment of the epicardial vessels, coronary microcirculation, and myocardium.

After STEMI reperfusion therapy, the culprit vessels already demonstrate patency. However, patients with STEMI have an instrumental injury to the epicardial vessels due to catheters, balloons, and stents, which may injure endothelial cells, causing the microthrombus to fall off, thus decreasing myocardial perfusion (20).

On the one hand, the decrease in resting MBF may be related to impaired microcirculation. Microembolization washout after thrombolysis treatment and transmural myocardial infarction may cause structural damage, leading to vascular obstruction (21). Moreover, interstitial edema, inflammatory cell infiltration, and platelet aggregation can also damage microcirculation patency (21). The effects of functional damage may occur after PCI, resulting in transient vascular contraction in epicardial vessels and microcirculation (22, 23). On the other hand, patients may have microcirculation injuries before STEMI, and most patients present with hypertension, smoking, hyperlipidemia, and diabetes, which may harm microcirculation (24, 25). Furthermore, microvascular structures may sustain further severe injury during subacute AMI through leakage of the red blood cells in blood vessels and leakage into the intercellular space of the myocardial body, forming intramyocardial hemorrhage (IMH) (26).

AMI mediates ischemia, and reperfusion causes edema of the myocardium, resulting in temporary impairment of cell function (27). A swollen myocardium and adjacent vessel compression may also affect blood flow patency. On the other hand, swelling may decrease tracer uptake, which further leads to the underestimation of resting MBF. In contrast, because of prolonged ischemia, some myocardium may undergo necrosis (26). Thus, there may be no or significantly reduced blood flow to the local myocardium.

Conversely, non-ischemic MBF is greater than ischemic MBF, possibly because the distal myocardium has a compensatory effect, resulting in increased resting MBF (28). However, this view needs to be confirmed by long-term imaging follow-up in the later stage.

### Resting MPI and resting MBF identity in the ischemic area

Most studies have paid less attention to the clinical significance of resting MPI in ischemic disease (29). However, only resting MPI combined with clinical features enables the diagnosis of obstructive CAD (30). In clinical application, patients with acute coronary syndrome (ACS) in the acute period cannot undergo stressing MPI (31). For STEMI, cardiologists use electrocardiogram and coronary angiography to locate the ischemic area. In our research, we applied resting MBF to identify ischemia. According to the ROC analysis, the AUCs were 0.666 and 0.671 with or without RPP correction. To some extent, MBF is able to identify residual ischemia in patients with STEMI.

### The influencing factors of resting MBF

In our study, the SRS and male sex were inversely related to the ischemic MBF.

**Figure 5:**
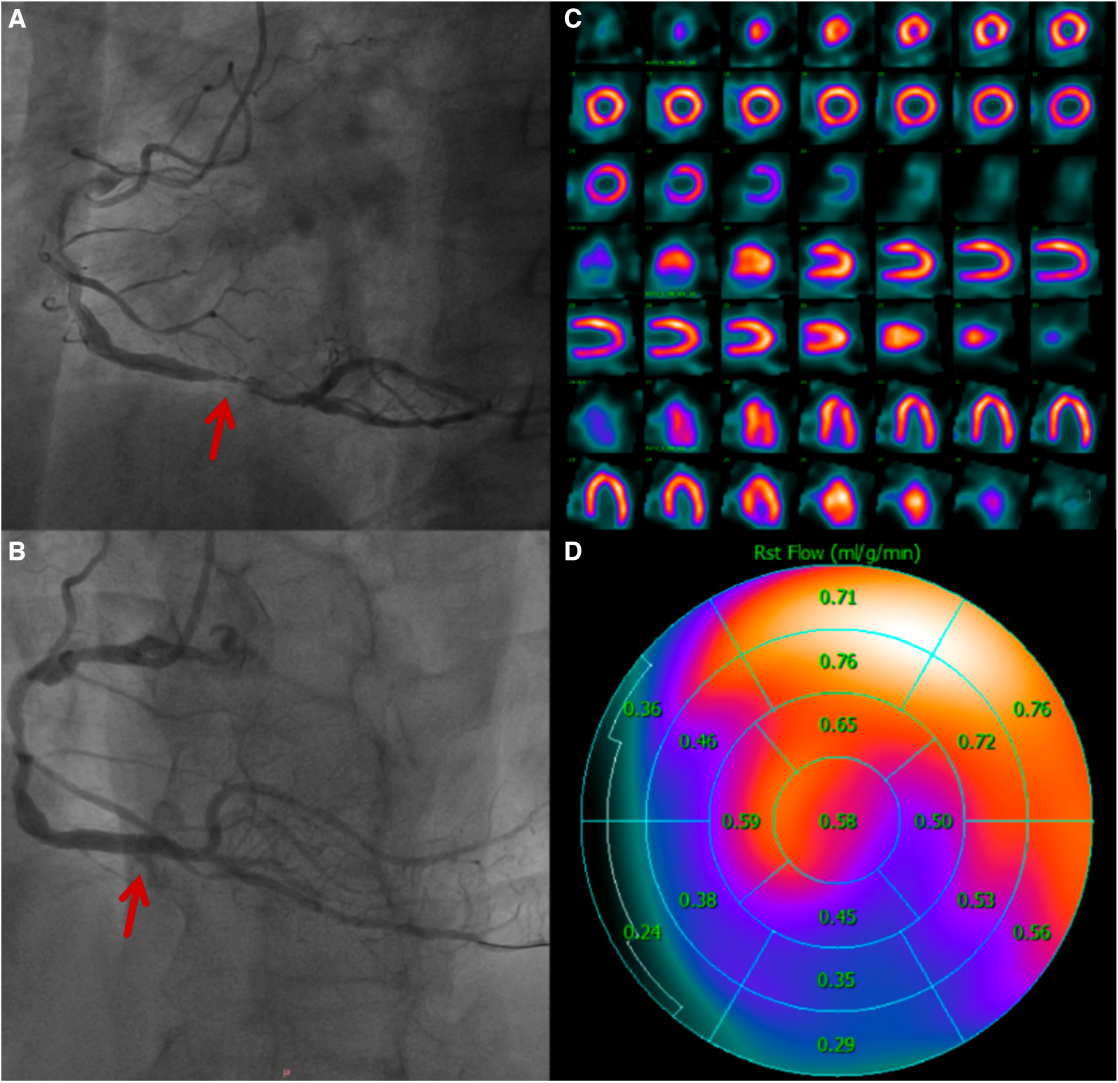
A 60-year-old man who was diagnosed with acute inferior ST-elevation myocardial infarction. 1.5 h after the onset of the disease, drug thrombolytic therapy was administered, the patient's clinical symptoms were alleviated, and his ST segment decreased by more than 50%. He entered the cath lab 11 h after the onset of illness. (**A**) CAG showed that the main lesion was located in the distal RCA posterior anterior trigeminal tubular, and approximately 95% of the stenosis and thrombosis was visible. (**B**) CAG after PCI, balloons placement and medication treatment. (**C**) After 4 days of treatment, there was normal in resting MPI. (**D**) Resting MBF showing that the RCA blood supply area was significantly lower than that in other regions.

The SRS indicates the degree of ischemia and infarct (32) and is often correlated with fixed ischemia. Previously, cardiologists and nuclear physicians mainly relied on SRS, TPD, and gated parameters to analyze the severity of myocardial infarction, prognostic information, and treatment decisions (9–12). Current CZT-SPECT systems enable the measurement of MBF, which is also proportional with regional myocardial perfusion (3, 33). SRS is derived from semiquantitative and visual analysis, which has limitations in multivessel diseases or balanceable ischemia (6). However, MBF, as a quantitative parameter, excludes the influence of human factors and is semiquantitative. It may be more sensitive for assessing and identifying ischemic areas than semiquantitative parameters (Figure 5). In our study, the higher the SRS, the lower the resting MBF. To some extent, ischemia-related MBF can reflect the severity of myocardial infarction.

Generally, male individuals have a greater risk of cardiovascular disease than female individuals (34, 35). Our study had the same characteristics as the general population distribution; male prevalence was greater, and female patients comprised only 9.33% of the study population. In a previous study, there was no significant difference in infarct size and cardiac impairment between women and men with AMI (36). However, the male sex was found to be an identical risk factor for coronary reduced flow and coronary microvascular disease (CMD) after myocardial infarction with thrombolysis (37). In our multiple linear analysis, the male sex had an inverse relationship with ischemic MBF.

### RPP influence

RPP and resting MBF are directly proportional, and currently available postprocessing software generally recommends RPP correction when measuring MFR. Moreover, some studies have also suggested that MFR without RPP correction also has prognostic value (38). In our study, only resting MBF was involved. There were statistically significant differences in ischemic MBF and non-ischemic MBF, both before and after RPP correction. In addition, there were small differences in the AUC and cutoff values before and after the correction. Furthermore, an excessively high heart rate and systolic blood pressure may overestimate resting MBF (15, 39), and we believe that RPP correction is optional for resting MPI only.

### Other factors

Resting MBF may also be affected by the degree of response to reperfusion treatment and individual differences.

### Limitations

The present study has some limitations. First, our assessment of myocardial infarction was performed after treatment, not before. Second, the study size was small. In the future, we can combine multiple medical centers to include more samples for research. Third, the long-term prognostic value of resting MBF was not discussed in this study, and we will follow patients and explore the prognostic value of resting MBF in patients with STEMI. Fourth, our study did not assess MFR, mainly because of the high risk of stress after STEMI. Finally, this study involved only the resting MBF and did not compare to the indices of microvascular resistance (IMR) (40, 41) and invasive resting flow (42). In the future, we will include a subcohort of patients with uncomplicated STEMI for MFR measurements. If possible, we will conduct comparisons of non-invasive resting flow and invasive resting flow to further investigate hemodynamics.

## Conclusion

In our small single-center study, resting MBF was able to test residual ischemia in patients with STEMI after reperfusion treatment and to identify ischemic area to an extent. Male sex and SRS had inverse corrections with ischemic MBF. A lower resting MBF may be associated with more severe myocardial ischemia. Further validation is expected in the future with a long-term follow-up.

## Data Availability

The original contributions presented in the study are included in the article/Supplementary Material, further inquiries can be directed to the corresponding author.
